# The Increasing Population Movements in the 21st Century: A Call for the E-Register of Health-Related Data Integrating Health Care Systems in Europe

**DOI:** 10.3390/ijerph192113720

**Published:** 2022-10-22

**Authors:** Arkadiusz Dziedzic, Abanoub Riad, Marta Tanasiewicz, Sameh Attia

**Affiliations:** 1 Department of Conservative Dentistry with Endodontics, Medical University of Silesia, 40-055 Katowice, Poland; 2 Department of Public Health, Faculty of Medicine, Masaryk University, 601 77 Brno, Czech Republic; 3 Department of Oral and Maxillofacial Surgery, Justus Liebig University, 35390 Giessen, Germany

**Keywords:** migrants, refugees, health-related data, central register, health care crisis, care provision

## Abstract

The escalating mass influx of people to Europe in the 21st century due to geopolitical and economic reasons as well as food crises ignites significant challenges for national health care services. The lack or disruption of cross-border, e-transferred, health-related data negatively affects the health outcome and continuous care, particularly in medically compromised individuals with an unsettled status. Proposal: The urgent need of a structured database, in the form of a health-related data register funded by the European Union that allows a swift exchange of crucial medical data, was discussed to flag ever-increasing migrants’ health problems, with a primary aim to support an adequate health care provision for underserved people who are at risk of deteriorating health. The data security information technology aspects, with a proposed and drafted structure of an e-health register, were succinctly highlighted. Conclusions: Focusing on long-term benefits and considering future waves of mass relocation, an investment in a health-related data register in Europe could vastly reduce health care disparities between minority groups and improve epidemiological situations with regard to major illnesses, including common, communicable diseases as well as oncological and infectious conditions. Commissioners, policymakers, and stakeholders are urged to continue a collective action to ensure vulnerable people can access health services by responding to the ongoing global migration crisis.

## 1. Introduction

### 1.1. The Crisis of Population Displacement in Europe

In light of the recent mass movements of people who have been migrating from African countries, such as Syria, Morocco, Algeria, Tunisia, and Sudan, and transiting across Europe, in addition to those who are relocating from Ukraine, many have experienced a restricted cross-border transfer of medical records that affects the individuals’ health status and health care provision for diverse underserved populations, resulting in the curtailment of health care [1,2,3]. According to United Nations High Commissioner for Refugees (UNHCR) data [4], the most recent conflict in Ukraine caused the displacement of more than 6 million people (updated 26 July 2022) across Europe, leaving them in peril of unsecure health care support. A vast number, 3,744,925 refugees from Ukraine, were registered for national protection schemes in Europe [4]. By the end of July 2022, the main directions of migration of Ukrainian refugees within Europe [4] were Poland (1,246,315), Germany (915,000), Czech Republic (400,559), Italy (154,710), Spain (130,160), France (92,156), Slovakia (87,027), Republic of Moldova (86,880), and Romania (84,384). The provision of critical services for Ukrainian refugees, with a special focus on health care, was prioritized in neighboring refugee-hosting countries. On the other hand, during the post-pandemic (COVID-19) period, there has been a continuous increase in first-time asylum applications in the EU member states and Norway, with a significant 28.2% increase in 2021, compared to 2020 [5]. According to the European Migration Network [5], the top five citizenships of first-time asylum applicants (EU and Norway) include Syria (98,895), Afghanistan (83,760), Iraq (25,995), Pakistan (21,020), and Turkey (20,400).

Amid the increasing influx of migrants and refugees, the broad sharing of vital information about individuals’ health between health care providers based in different countries should be considered an integral element of the global public health response to secure basic human rights, regardless of origin and ethnicity [6]. A current situation in East Europe [7] revealed that uninterrupted e-access to medical records in all European countries would provide cost-effective support that may, in some instances, save lives. Populations of migrants with unmet multifactorial and multifaceted health needs [8] tend to turn to community support and self-management of medical issues with unpredictable consequences. These increasing challenges are alarmingly exacerbated in undocumented migrants [9]. 

A great opportunity exists for authorities and commissioners to promptly implement modern digital technologies tailored specifically to helping refugees resolve their health-related problems and to supporting health care services deliver a broad range of comprehensive care across the continent for persons fleeing from areas of conflicts. The joint political and public health efforts to mobilize the decision-makers to address the unmet health needs of refugees are urgently required while coping mechanisms employed to tackle the crisis need further enhancement. Reportedly, because of the constant influx of forcibly misplaced people to the European continent, health care providers are convinced that ad hoc, not a structured health care provision, is not sufficient [3,10]. Ultimately, an e-transformation within the European health care systems by profound integration of their information technology (IT) infrastructure can make a real difference to underserved and underprivileged people [11,12].

### 1.2. Transiting Borders by Vulnerable Individuals Who Require Health Assistance and Complex Care

**Figure 1:**
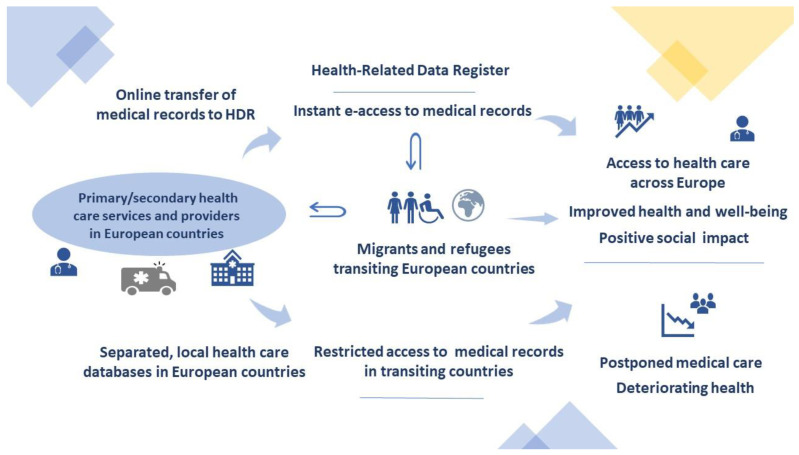
The implications of the introduction of a European register collecting health-related data and its impact on refugees’ well-being.

Since refugees tend to cross multiple borders within the continent of Europe before obtaining a settled status, assuming that they can seek some form of medical care in transiting countries, a smart electronic medical data transfer between health care providers and health boards operating in specific healthcare systems (public/private/mixed) would allow effective and timely diagnostic regimes, treatment, diseases monitoring, specialist consultations, and vaccination status check. This particularly implies the continuation of complex therapeutic protocols by multidisciplinary medical teams from different countries. The enhanced e-access to such data by hospitals, general practices, medical institutes, university hospitals, and any other health care providers on various levels of reference will inevitably improve the medical information transfer and, as a direct consequence, will facilitate the optimal healthcare, regardless of public health systems variations (Figure 1).

## 2. A Central Electronic Register Integrating Health-Related Data in Europe

European countries who have agreed to support and host refugees, allowing a relocation of thousands of people, are obliged to provide adequate health/dental care on multiple levels for refugees fleeing from their country of origin, particularly children [13,14]. It has been reported that refugees experience numerous problems, especially accessing specialist health care [15]. Therefore, the concept of a health-related data e-register (HDR) in Europe, comprising medical/health records, medical history, treatment protocols, diagnostic results, pharmacotherapy, and vaccination details for displaced migrants with an unsettled status could substantially underpin various health care systems and enable clinical data to be shared across the continent. This initiative encapsulates well and aligns with the World Health Organization (WHO) policies as a part of the strategy for supporting systems worldwide to achieve ‘universal health coverage’ [16] and inclusive primary healthcare. The most recent report of WHO [15] *“demonstrated critical gaps in data and health information systems regarding the health of refugees and migrants and they are fragmented and not comparable across countries”.* It can be clearly noted that migrants’ health statistics are often missing in global datasets used for public health monitoring. Reportedly, such data collection and monitoring systems should guide effective interventions among diverse groups of migrants to reduce health outcome disparities and address their health needs [13,17].

The main purpose of the central register deployed collectively by European countries to address the immigration crisis aims to enable secure, real-time access to crucial clinical information. It is expected that an inclusive e-register designed to integrate various health-related datasets, functioning as a clinical data sharing e-warehouse, intends to collect and store clinical information across different health care services [18,19] to be a core part of the safety e-network to improve the quality and continuity of care as well foster research projects. Preferably, an HDR should incorporate already existing internally-funded clinical/research registries in European countries and similar external databases outside the European Union (EU) governed by others, e.g., WHO. The inter-relationship among them must be clearly specified, with strict legislative measures for personal, sensitive data protection. While a most recent pandemic enabled a mass collection of information about billions of individuals during COVID-19 vaccination programs, the big data associated with the introduced EU digital vaccine e-passport might underpin the success of a central e-register launch campaign. By default, such an e-health database that stores information from medical services needs to be distinguished from other services, particularly those belonging to the social care sector. Whilst the existing state-based e-registries in EU and non-EU countries utilize heterogeneous IT infrastructures, the vast majority of them are not deemed compatible. It is assumed that currently functioning non-uniform digital systems across Europe, with different levels of electronic protection, are susceptible to data security breaches.

The crucial coded data contained on the central European e-register should include patient’s identifiers (patient’s full name, date of birth, nationality, contact information—if applicable, next of kin, emergency contact information, insurance, medical record number, i.e., unique patient’s ID), demographic information (age, gender, ethnicity, marital status, education), social data (occupation, smoking status, employment, socio-economic status, housing conditions), unstructured clinical notes, vital signs, encoded and standardized coded diagnoses (International Classification of Diseases, Diagnostic and Statistical Manual of Mental Disorders, Systematized Nomenclature of Medicine), pharmacotherapy (generic names, National Drug Codes, Systematized Nomenclature of Medicine, RxNorm), procedures (primary care procedures, minor and major surgery, operative procedures, radiology, pathology, diagnostic procedures), and additional and laboratory results (tests) [19,20,21]. In the light of a modern inclusive approach toward minority populations, disclosing information about race/ethnicity background does not seem necessary, and only the basic personal details, along with medical data, should be prioritized during the data uploading/input process. Arguably, the group of ‘undocumented’ migrants and refugees without valid proof of their personal data, who fail to secure their identity, can also be eligible for the e-registration process following a cross-check and further verification within the international databases once they obtain an identification document/national identity number. The process of digital e-health account registration to obtain valid, informed consent must be robust, transparent, and, more importantly, voluntary, with the unconditional option to withdraw consent at any time. This process may require a suitable level of ‘digital competency’ in persons involved (a potential limitation). Hence, the multilanguage, professional interpreting services are intended to play an essential role while translating the register-related terms and conditions.

As the primary e-hub in Europe, an HDR appears especially useful as a source of information to manage potentially life-threatening diseases, oncological conditions, rare genetic disorders, and severely infectious diseases [22]. The development of the interoperable e-health hub is expected to be linked with existing registries designed for public health data collection and biomedical research. This approach is already reflected in the long-term strategies of the e-health agenda of the EU Commission [23] while the main priority of the digital transformation of health is “*to secure citizens’ access to their health data, including across borders, enabling citizens to access their health data across the EU*” [24]. Predictably, the EU e-health policy will address the anticipated migration crisis that is likely to arise as a consequence of geopolitical instabilities and food shortages.

**Figure 2:**
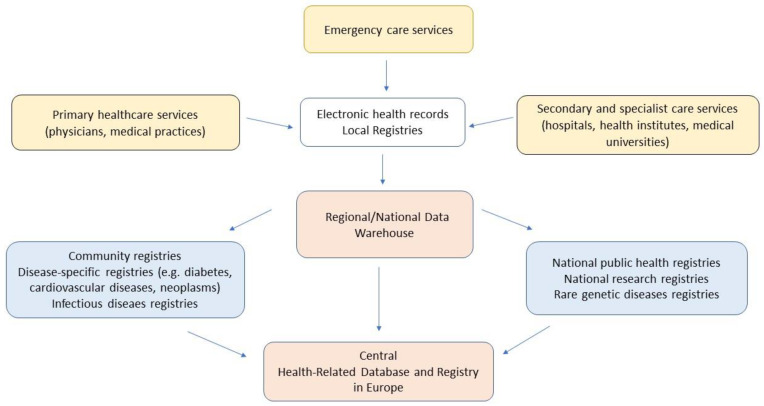
The proposed multilevel structure of the conceptual integrated network of registries containing health-related data (own interpretation, based on [19]).

Fundamentally, the EU is expected to provide a firm legislative basis, hardware, software, and maintenance of the HDR. A proposed HDR network and recommended infrastructure is presented in Figure 2. Noticeably, a customized, comprehensive IT architecture of country-based health registries that act as gateways to the HDR are necessary elements of its connectivity and functionality.

In fact, the concept of a centralized e-health database can be particularly important considering the ongoing COVID-19 pandemic, new waves of SARS-CoV-2 or other variants outbreaks, and the vaccination status of a huge number of misplaced and relocated people crossing European borders. The exchange of crucial medical/public health information, including medical history, such as primary medical conditions, allergies, medications taken, vaccination status, and/or tests results, is paramount during an unprecedented large movement of populations in the 21st century.

In July 2022, the World Health Organization (WHO) announced a ‘report on the health of refugees and migrants’ [17], stating that “*many migrants and refugees face poorer health outcomes than the host populations*” and “*addressing their needs is, therefore, a global health priority and integral to the principle of the right to health for all*”. As currently available evidence on the health of these groups appears, unfortunately, insufficient, the “*comparable data across countries and over the time are urgently needed to track progress towards the health-related United Nations Sustainable Development Goals (UNSDG) 2030*”. The idea of an HDR meets this such recommendation as it resembles the main goal of the United Nations Sustainable Development, i.e., good health and well-being (goal 3) [25].

Additionally, actively supporting European countries declare considerable challenges in obtaining relevant documentation and barriers in health-related data sharing with other health care providers abroad, which is deemed highly restricted or frequently impossible. Inevitably, considering the dynamic scenario associated with the mass transrelocation of people via multiple countries, the real-time, uninterrupted access to health-related information could facilitate a decision-making process regarding the diagnosis, therapy, and preventive measures for primary and secondary health care services. The concept of electronic cross-border health services and e-health digital service is deemed a priority for the European Commission [26]. EU member states have already use large-scale digital technologies, online platforms, electronic registration systems, and new remote procedures for border migration management, including the Enter−Exit System and European Travel Information and Authorization System. Expectedly, this IT infrastructure could support the implementation of a health-related data register. The usefulness of a central HDR is presented in Table 1.

### Potential Constraints and Implications of Broad Online Data Sharing

The fundamental problems associated with the health-related data register development and its practical implementation include [27,28,29] informatics aspects, adequate IT infrastructure and expertise of health care providers, IT digital security issues, personal data protection (de-identified vs. identifiable), patients’ privacy, ethics, exposure to cyber-attacks, language discrepancy, informed consent, missing medical documentation, lack of identifying documents, and an unknown status of a young person below the age of 16 without an accompanying person possessing a legal responsibility towards the child. The question arises whether health care providers require a valid consent from the individual to receive access to the personal record. This aspect must be prioritized when establishing standard operating procedures for the HDR to function, considering the risks and benefits rate, with the time factor having a significant impact in allowing instant access to an individual medical record. Countries involved in rescue missions helping refugees, guided and supervised by local and European Union authorities, should be responsible for the digital data safety storage and international e-transfer, if applicable. The rules related to health-related data sharing require a strict regulatory governance, which can be challenging as a result of the variation in national health care systems. Undoubtedly, personal and sensitive medical data protection prevails over the other secondary aspects, underpinned by a structured legislative framework that is supposed to specify the criteria for persons who are eligible to access database, predominantly health care workers and clinical medical/dental personnel directly involved in patients’ care. 

Ultimately, the security of digital ‘big data’ of individuals’ health status can be additionally enhanced by using highly encrypted data/files, a virtual private network, peer-2-peer systems, and a multilevel verification of the patient’s identity as a genuine account holder. Arguably, the adoption of the newest digital security certificates for medical records and documentation (radiographs, tests results, etc.), such as non-fungible tokens, as well as the cryptographic assets on the blockchain technology with a unique identification code and metadata [30,31,32] will allow users to distinguish and recognize health data attributed to individuals. Equally, the healthcare provider accessing the HDR should be carefully verified prior to using the database containing sensitive data. In fact, the maintenance, updating, security tools, and scaling up of the HDR compose the main challenges. The unlimited and unrestricted online access to personal e-health account determines the likelihood of the e-health register’s successful implementation and digital data broad distribution among health care services. In addition, the other, non-IT-related reasons may preclude and restrict the migrants’/refugees’ e-registration, including fear of deportation, abscondence, and stigma as well as cultural, religious, and gender identity issues. These factors equally apply to avoidance of medical/dental treatment in the first place in people translocated from various cultural backgrounds. Lastly, the expected successful launch of a health-related e-register depends on the relevant campaign reaching minority groups and supplying sufficient information regarding the unprecedented benefits of modern digital data exchange to secure individuals’ health. 

The process of setting up a ‘pan-European’ e-health register requires joint efforts of committed organizations and cost-effective measures to capture complex, comprehensive medical data, collect adequate information in a safe manner, and share them between involved HCPs. Safeguarding data security, especially sensitive information regarding medical status, therapies, and genetic profile, are deemed the essential constraint of the central e-register development and workflows. 

## 3. Proposed Action to Initiate a Central European E-Health Database Rollout

The recommended steps allowing the implementation of a central e-register, applicable for both public and private health care providers, include:

1. Wide-scale upgrade of currently functioning health registries and e-systems in European countries on local, regional, and national levels.
2. Identification of crucial, compatible IT infrastructures to allow a unification of data exchange.
3. EU legislation approving a centralized health-related data storage system, with an open collaboration of all European countries, also not formally members of European Union.
4. Allocation of individual health care numbers allowing the access to public health care for persons with formal migrant/refugee status.
5. Implementation of individual e-health account and/or mobile appt, including copies of medical records (translation options).
6. Integration of local health care providers’ databases linked to the EU; legally approved and commissioned central online storage database of healthcare records. Data can be imported and exported between EU countries.
7. Optimization and standardization of e-data exchange between countries based on advanced IT infrastructure.

On a local level, we urge governments to develop/extend secured medical data e-storages that are protected from cyber threats and allow swift data exchange and communication between health care providers functioning in different national public health systems. This should be executed in the context of local and national priorities for funding and developing health care services, responsible for both acute/emergency and elective care. Practitioners and professionals are expected to fully engage while using the centralized health-related data register, taking patients’ needs, preferences, and values into account. Other humanitarian organizations, such as United Nations Refugee Agency, UNICEF, and Doctors Without Borders, may actively solicit data exchange and external support.

## 4. Conclusions

The current pending conflicts in Africa, the Middle East, and Eastern Europe as well as an increasing risk of escalation in the Balkans region have accelerated the global efforts of considering the management of healthcare needs of misplaced people and reflected discrepancies in European health care systems. The mass influx of people triggered concerns among public health services about unequal access to health care services because of a lack of medical e-data sharing between non-compatible IT infrastructures. Providers across Europe outlined critical public health resources, such as a safe exchange of crucial data navigated by a central e-register, to enable its international integration.

As humans’ health and well-being is an utmost priority, only joined international efforts, firmly backed by financial resources, can succeed in an unstable area of mass peoples’ relocations. Western governments, regardless of their specific political agendas, are required to protect individuals’ well-being and health amid recurrent refugees’ crises. Equally, health care providers have a responsibility to enable and secure data transfer to improve health care outcomes. Individuals holding migrant and refugee status have a legitimate right to receive high quality, integrated health and social care services to ensure coordinated care across regions. Innovative strategies that seek to reach out unsettled populations are urgently needed to mitigate ever-increasing health disparities.

## Figures and Tables

**Table 1:** Expected benefits of a central health-related data register governed by the European Union Council and associated institutions.

Reduction of health care inequalities by enabling access to individuals’ medical records by any health care providers to facilitate efficient care, urgent and elective treatment, and diagnostic measures. Instant data exchange between various health care services in different countries. Coordination of complex care for underserved populations. Support for local authorities, commissioners, and health care providers in primary, secondary, and tertiary sectors. Opportunity to set up international network/council of medical professionals consulting most challenging cases. Medical supply and medical aid support on an international scale. Environmentally sustainable health care network. Elimination of unlawful discrimination in accessing health care services. Impetus and incentive for epidemiological and biomedical research. Fostering access to big data sources, boosting research of health-related projects. Prevention of human trafficking. Active surveillance of vaccination/testing and infectious diseases data. Verification tool for person’s identity. High level of digital security of confidential data.
